# The Role of NADPH Oxidase in Neuronal Death and Neurogenesis after Acute Neurological Disorders

**DOI:** 10.3390/antiox10050739

**Published:** 2021-05-07

**Authors:** Song Hee Lee, Minwoo Lee, Dong Gyun Ko, Bo Young Choi, Sang Won Suh

**Affiliations:** 1 Department of Physiology, College of Medicine, Hallym University, Chuncheon 24252, Korea; sshlee@hallym.ac.kr (S.H.L.); minwoo.lee.md@gmail.com (M.L.); kdkhiah@gmail.com (D.G.K.); bychoi@hallym.ac.kr (B.Y.C.); 2 Department of Neurology, Hallym Neurological Institute, Hallym University Sacred Heart Hospital, Anyang 14068, Korea

**Keywords:** NADPH oxidase, neuronal death, neurogenesis, stroke, traumatic brain injury, epilepsy, hypoglycemia

## Abstract

Oxidative stress is a well-known common pathological process involved in mediating acute neurological injuries, such as stroke, traumatic brain injury, epilepsy, and hypoglycemia-related neuronal injury. However, effective therapeutic measures aimed at scavenging free reactive oxygen species have shown little success in clinical trials. Recent studies have revealed that NADPH oxidase, a membrane-bound enzyme complex that catalyzes the production of a superoxide free radical, is one of the major sources of cellular reactive oxygen species in acute neurological disorders. Furthermore, several studies, including our previous ones, have shown that the inhibition of NADPH oxidase can reduce subsequent neuronal injury in neurological disease. Moreover, maintaining appropriate levels of NADPH oxidase has also been shown to be associated with proper neurogenesis after neuronal injury. This review aims to present a comprehensive overview of the role of NADPH oxidase in neuronal death and neurogenesis in multiple acute neurological disorders and to explore potential pharmacological strategies targeting the NADPH-related oxidative stress pathways.

## 1. Introduction

Acute brain injuries, such as strokes, traumatic brain injury (TBI), epileptic seizures, and hypoglycemia-induced damage, all result in a substantial disease burden for both individuals and society at large. Though these injuries may have different neurological manifestations, a common pathophysiological process is cellular oxidative stress, which is largely induced by the excessive production of reactive oxygen species (ROS). For the past few decades, many studies focused on the role of ROS-generating pathways in various neurological disorders and led to the identification of nicotinamide adenine dinucleotide phosphate (NADPH) oxidases as one of the major sources of ROS. While NADPH oxidase (NOX) contributes to normal physiological processes in the central nervous system, excessive activation of NOX results in neuronal damage in the setting of acute brain injury. In this current review, we summarize the evidence supporting the pathophysiological roles of NOX after acute stroke, TBI, epileptic seizure, and hypoglycemia. In addition, we explore the importance of NOX balance regarding the neurogenesis process after such brain injuries.

## 2. Nicotinamide Adenine Dinucleotide Phosphate (NADPH) Oxidases (NOXs)

The NOX family consists of membrane-bound enzymes that transport an electron from NADPH to oxygen, thereby producing superoxide free radicals [1]. In this process, oxygen (O_2_) is transported to the intracellular space, and hydrogen (H+) is exported to the extracellular space. Hydrogen peroxide and ROS, including hydroxyl radicals, are also produced as a result of this reaction. The superoxide produced by NOX plays an important role in killing foreign substances, but excess superoxide can lead to oxidative stress and cell damage. Numerous studies have shown that the NOX system is responsible for the production of ROS [2,3,4]. The NOX family consists of several members, including the catalysts of the complex: NOX1, NOX2, NOX3, NOX4, NOX5, dual oxidase 1 (DUOX1), and DUOX2 [5,6,7,8,9]. Once the active complex is assembled, an electron from cytosolic NADPH is transferred to oxygen on the extracellular side [1]. The distribution and regulation of the NOX and DUOX isoforms throughout the body and brain vary depending upon the isoform.

Oxygen (O_2_), which must be supplied to the living organism, is an essential molecule for survival. However, the fact that oxygen induces oxidative stress in metabolic processes in vivo and that the ROS produced therein damages cells or tissues has already been confirmed through many studies. In vivo oxidative stress is a phenomenon that occurs when the balance of antioxidants and oxidants in cells or tissues is disrupted. Oxidative signaling is reported to protect the biological response in healthy cells by acting as a secondary messenger and playing a role in many cell signaling pathways. However, oxidative stress occurs when reactive oxygen species are formed excessively or when the concentration of antioxidants is low, resulting in oxidative injury. Oxidative stress generated by NOX is considered to be a secondary effect rather than the primary cause of disease; however, it can have a powerful effect on magnifying the resulting damage suffered as a consequence of a given injury. Oxidative stress has been reported to be involved in diseases, such as inflammatory disease; hypoglycemia; cardiovascular diseases, such as stroke; and neurodegenerative disease, including Parkinson’s disease and Alzheimer’s disease. It has been reported that, in these diseases, neurological outcomes are improved through treatment with antioxidants, post-injury. Based on these findings, we describe the mechanisms responsible for the generation of ROS due to NOX in vivo and the sequential neuronal death. We also describe how NOX activity is associated with neurogenesis after acute neurological illness.

## 3. NOX Induced Oxidative Stress and Subsequent Neuronal Death

The phenomenon of oxidative stress, which occurs due to imbalance between ROS and antioxidants, is widely investigated as one of the major pathophysiological causes of neurological injury [10,11,12]. The most commonly occurring cellular free radicals promote the formation of other ROSs, leading to lipid peroxidation [13,14]. Several studies have demonstrated that NOX is elevated after brain damage, such as traumatic brain injury (TBI), stroke, epilepsy, and hypoglycemia [15,16,17,18]. Pharmacological and genetic inhibition of NADPH oxidase has been shown to significantly attenuate secondary neuronal damage, suggesting that NOX plays an important role in the onset and progression of the pathology [19,20]. It is known that ROS increases along with NOX, and the degree was found to be closely correlated with the patient’s prognosis. Thus, attempts to improve the prognosis by neutralizing reactive oxygen species via the administration of antioxidants to the patient naturally followed [21]. 

Among the various mechanisms of neuronal injury, neuronal excitotoxicity is one of the earliest cascades induced after acute brain injury. Excitatory toxicity is primarily due to the persistent action of glutamate on the neuronal glutamate receptor, an N-methyl-D-aspartate (NMDA)-type glutamate receptor [22,23]. Excitotoxicity is a particularly important pro-apoptotic mechanism in stroke [24]. Nitric oxide is nonpolar, lipid permeable, and has a relatively long half-life and diffusion distance in the brain. Nitric oxide is produced by neuronal nitric oxide synthase in response to Ca^2+^ entry through the NMDA receptor. Although the cause and regulation of NMDA-induced peroxide production is unclear, increasing evidence points that the dominant cause is neuronal NOX in this environment [25].

**Figure 1:**
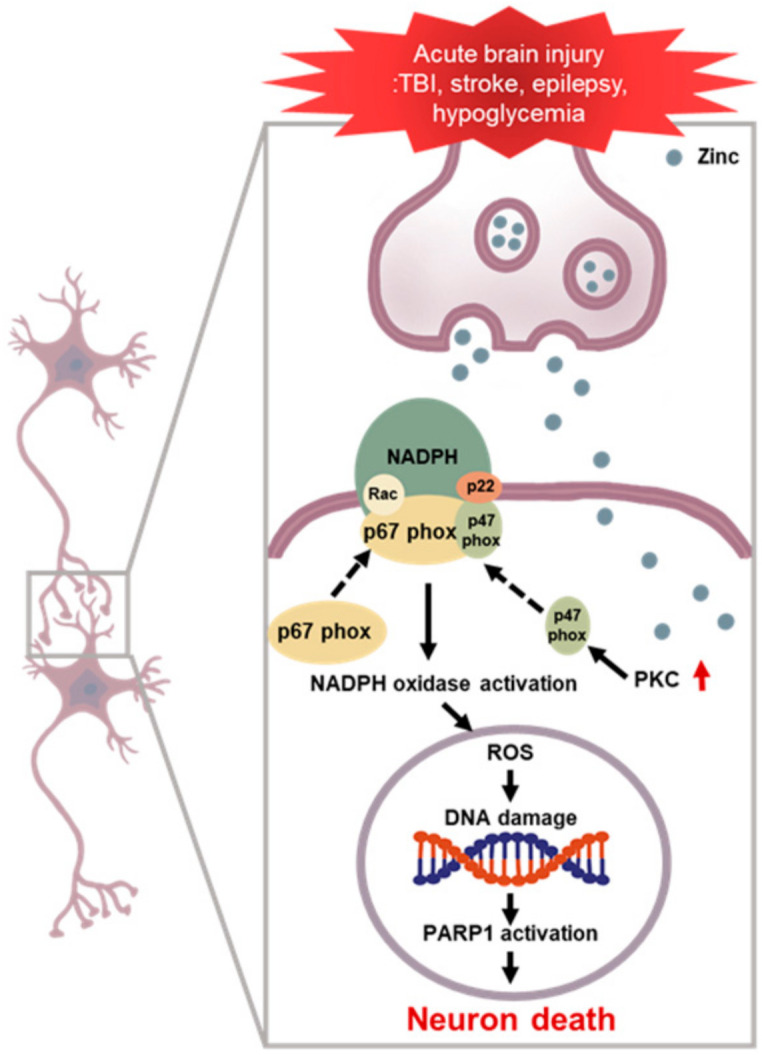
NADPH oxidase induced neuronal death after brain injury. Multiple acute neurological disorders lead to zinc release from the presynaptic neurons and translocation into the postsynaptic neurons, which, in turn, activates neuronal NOX. The p47phox component is phosphorylated and migrates to the plasma membrane, where it binds with other subunits to form an active enzyme complex. Activated NOX produces ROS, which is released into the intracellular or extracellular space and contributes to progressive DNA damage. ROS produced by NOX lead to further accumulation of zinc in the postsynaptic neurons, aggravating the cascades. PARP 1 is excessively activated due to DNA damage and leads to energy failure and mitochondrial dysfunction. This devastating cascade eventually leads to neuronal death.

Free zinc is known to mediate NOX activation and exacerbate neuronal death. There is a high concentration of zinc in neurons [26]. Since 1980, zinc has been known to be present in synapses and to be released into the synapse in response to depolarizing activity of neuronal cells and subsequently regulate various ion channels through specific zinc-binding domains. It has also been confirmed that some of the synaptically released zinc flows into postsynaptic neuronal cells and regulate intracellular signaling [27,28]. Synaptic zinc is a neurotransmitter and is thought to act as an intracellular signaling molecule. In addition to its physiological action at these synapses, zinc contributes to several pathologies. An excessive influx of zinc and excessive accumulation of zinc in postsynaptic neurons lead to neuronal death. Our previous studies have revealed that the influx of zinc is an important mechanism of neuronal cell death in neurological disorders, such as cerebral infarction, traumatic brain injury, hypoglycemia, and epilepsy [29,30]. When the concentration of zinc in the cell increases rapidly, various oxidative damage processes are known to be induced in the accumulation of zinc in the mitochondria and the activation of NOX. Oxidative damage, in turn, activates an enzyme called PARP, depleting energy metabolites in the cell and eventually leading to cell death [31,32]. PARP is an important protein in the regulation of cell death and is a marker of apoptotic and cell death. The transient increase in PARP activity with mild injury facilitates the DNA repair process. However, with excessive damage, prolonged activation of PARP leads to excessive depletion of NAD and ATP levels within the cell, leading to cell death [33,34,35,36,37]. If PARP is activated by zinc, it could be assumed that increased intracellular zinc levels are associated with increased PARP activity [38]. It has been reported that zinc overload leads to an increase in intracellular ROS in an NADPH oxidase-dependent manner, suggesting that ROS production may contribute to zinc-induced PARP activation [29] (Figure 1). When PARP levels were measured to further investigate the effect of zinc on apoptosis, PARP levels increased significantly over time after damage, which was significantly decreased by treatment with TPEN and EDTA, a chelator with a high affinity for zinc [15,16,39]. Since NOX is activated when the active complex is assembled, many studies have investigated the possibility of NOX as an effector against oxidative stress [29,40]. NOX inhibitors have been researched and developed to counter the harmful effects of NOX. The NOX complex consists of the independent cytoplasmic subunits p47-phox, p67-phox, p40-phox, and Rac and membrane subunits gp91-phox and p22-phox, and it requires a unique activation process mediated by subunit interactions [41]. Therefore, it is possible to prevent neuronal cell death by blocking NOX that has been activated due to neuronal damage or inhibiting subunits that constitute NOX. Among these subunits, p47-phox plays the most important role in constructing complexes for NOX’s activation, and function by binding the cytoplasmic subunit, and translocating it to the membrane and anchoring to p22-phox [42]. When systemic inflammation was induced through lipopolysaccharide (LPS) in gp91phox-/- (knockout) and p47phox-/- (knockout) mice in an in vivo experiment, the inflammatory response was significantly lower in the brain (including hippocampus) of gp91phox and p47phox knockout mice than in wild-type (WT) mice [43]. In addition, p47phox-deficient neurons produced significantly less peroxide after hypoglycemia and had reduced neuronal death than WT neurons because neurons from p47phox-deficient mice were unable to assemble the active NOX complex [39,44,45]. Apocynin, an inhibitor of NOX, has been reported to have a neuroprotective effect in various chronic central nervous system disease. Cell viability was increased by reducing the release of pro-inflammatory cytokines, such as TNF-alpha and IL-1 beta [46,47].

### 3.1. Stroke

#### 3.1.1. NOX Induced Neuronal Death after Stroke

The main pathological elements of both types of stroke, ischemic and hemorrhagic stroke, are oxidative stress derived from an impaired supply of blood, oxygen, and glucose. In ischemic stroke, ROS are generated in a local hypoxic lesion, as anaerobic metabolism dominates. This process includes the free radical superoxide, primarily generated not from the uncoupling of nitric oxide synthase (NOS) but from NOX and mitochondrial enzymes. ROS also disrupt the blood–brain barrier, inducing cytokines, such as metalloprotease and also NF-kB via vascular endothelial growth factor (VEGF) expression. These inflammatory reactions also lead to the generation of ROS, worsening the penumbra region [20,48]. Thus, it is suggested that NOX may be intertwined with neuronal death in ischemic stroke. Activated microglia and astrocytes were observed in the ischemic stroke animal models and were known to produce peroxides through NOX. Activated microglia can also promote the release of inflammatory factors, such as IL-1β and TNF-α, and NOX2 inhibition reduces the activation and proliferation of microglia, and activation of NF-κB, resulting in less oxidative stress and inflammation [49,50].

During intracerebral hemorrhages (ICH) and subarachnoid hemorrhage (SAH), activation of NOX is detected. Immunoreactivity to the gp91phox subunit was found to be increased during both ICH and SAH models, especially NOX2 [32,51]. In addition, its activation leads to sequential damage as vasospasms, worsening the pathophysiological processes [52]. Notably, blood influx causes an abnormal increase in calcium ions as NMDA receptors are activated. This results in NOX and the mitochondrial electric chain producing more ROS, damaging the brain [53]. Although NOX2 knockout mice did not show improved outcome after SAH [54], hemorrhage does activate NOX and contributes to increasing the magnitude of oxidative stress.

#### 3.1.2. Neuroprotective Effect of NOX Inhibition on Stroke

Considering that NOX is essential for the production of ROS and leads to brain cell damage in various types of stroke, controlling the activation of NOX was thought to minimize neuronal damage from oxidative stress. NOX inhibition was experimentally achieved by either the implementation of NOX-related gene knockout mice or the administration of apocynin, a selective NADPH-oxidase inhibitor with IC50 of 10 μM [55]. Numerous studies showed that inhibition of NOX families reduces the damage from stroke. Although the major protective effects are from NOX 2 or NOX4 inhibition, a study with NOX1 inhibition by Adeno-associated virus (AAV) containing NOX1 short hairpin RNA (shRNA) showed decreased peri-infarct after stroke. The mice were given 90 min middle cerebral artery occlusion (MCAO), and the size of infarcts was measured by Nissl staining. It was proved that the number of dead neurons and activation of astrocytes were decreased with NOX1 inhibition using NeuN and TUNEL staining [56]. Ischemic–reperfusion damage is of clinical significance in ischemic stroke. This includes both oxygen and glucose deprivation from the impaired blood circulation. According to our previous study, NADPH oxidase is activated as hypoglycemia provides substrates, increasing oxidative stress. Inhibition of NOX with apocynin (15 mg/kg) yielded lowered ethidium fluorescence, thus proving the neuroprotective role of NOX deactivation in acute stroke [57] (Table 1).

### 3.2. Traumatic Brain Injury 

#### 3.2.1. NOX Induced Neuronal Death after TBI

Traumatic brain injury not only includes primary mechanical injury but also secondary neuronal injuries in chronic stages. Primary neuronal injury results in inflammation spreading of cerebral damage, disruption of the BBB, and sequential neuronal damage. Under such circumstances, ROS is produced and deteriorates the viability of neurons. NADPH oxidase activation was observed 1 and 24–96 h after TBI, parallel to the rise of ROS. In a study examining only late time period (24 to 48 h) after TBI, it was confirmed that NOX2 activation in microglia significantly contributed to neuronal damage after TBI at 24 to 48 h, and NADPH oxidase activation increased ROS, as observed in the study. The likely cause would be the scavenging of ROS production by the administration of the NADPH oxidase inhibitor, apocynin [7]. The production of ROS is also intertwined with NOX, as NOX is activated during secondary damage of TBI. Thus, NOX inhibition has shown to decrease neuronal damage after TBI [58]. Two peaks of NOX activity are noted after TBI. The first peak is within one hour after the trauma, involving NOX2 in neurons. The secondary peak is 24–96 h after the trauma. Although the origin of ROS production in this peak remains unclear, it involves NOX activation in microglia. Previous studies have demonstrated that NOX2 is confined to neurons and microglia in the cerebral cortex and hippocampus [1,59]. NOX is also confined to the microglial, so the extracellular ROS produced by microglia is directly toxic to neurons, possibly contributing to neuronal cell death [60]. The result with beta-amyloid suggests that NOX is also associated with post-traumatic dementia, with the involvement of decreased activation of microglia [7]. A postmortem study showed the activation of NOX2 and NOX4 around 6 to 24 h after the trauma, and their activation was higher in patients with a worse Glasgow coma scale score [19]. Therefore, inhibition of NOX after TBI may have a therapeutic potential for neuroprotection.

#### 3.2.2. Neuroprotective Effect of NOX Inhibition in TBI

NOX inhibition has shown a neuroprotective effect after TBI in the hippocampal region. Our previous studies demonstrated this with the induced TBI model using mice and 24 h of apocynin injection (100 mg/kg). Detecting the oxidative stress with 4HNE staining showed that the inhibition of NOX led to an increased survival rate of pyramidal cells. The result was consistent with results of BBB disruption and microglial activation responsible for increased ROS [61] (Table 1).

### 3.3. Epilepsy

#### 3.3.1. NOX Induced Neuronal Death after an Epileptic Seizure

Epilepsy can have genetic causes or result from acquired neurological injuries, such as a traumatic brain injury, infection, or stroke, in a process known as epileptogenesis. In genetic epilepsy, a variety of pathogenic variants in genes governing ion channel, mitochondrial, and endogenous antioxidant systems may predispose individuals to developing the disease. In previous studies, with diverse models to silence or overexpress the endogenous antioxidant response, involving agents, such as thioredoxin, oxidative stress was seen to be a crucial part of epileptogenesis. Epilepsy activates astrocytes and microglia, making them hypertrophic and reactive. Reactive glial cells release pro-inflammatory mediators, which, in turn, act on neurons, increasing the expression of redox-sensitive transcription factor activating protein -1 (AP-1) and NFκB, thereby activating NOX in microglia. Activation of NOX in microglia increases the production of extracellular peroxide via iNOS. These factors can affect neuroblasts or neurons that have recovered after epilepsy [62]. In acquired epilepsy, changes in neurotransmitter receptors and ion channels induced by oxidative stress exacerbate the symptoms. The main pathophysiology is associated with excitotoxicity and apoptosis [63]. The main sources of ROS production in epilepsy are known to be mitochondrial dysfunction and the activation of NOX and xanthine oxidase (XO). However, NOX and XO activation was seen to be significant in a calcium-ion-independent manner, in which mitochondria cause a calcium ion influx. The study examined the inhibition of NOX and XO and showed that neuronal hyperexcitability is caused by ROS production from NOX and XO [64,65]. A correlation between NOX2 and seizure was also revealed in the pilocarpine-induced seizure model. When temporal lobe epilepsy (TLE) develops, NOX2 is activated [66]. Therefore, controlling ROS levels with the inhibition of NOX would appear to have therapeutic importance. 

#### 3.3.2. Neuroprotective Effect of NOX Inhibition on Epileptic Seizure

Both reducing neuronal death and maintaining the integrity of the BBB are important features for neuroprotective agents in the context of oxidative stress. Notably, NOX inhibition can positively influence such variables [67]. NOX inhibition with apocynin (30 mg/kg) has showed that the decrease in oxidative stress resulted in less neuronal death from a seizure. A study evaluating ROS production with dihydroethidium in a model of TLE showed that apocynin reduces ROS production, oxidative injury, BBB disruption, neutrophil infiltration, microglia activation, and neuronal death in vivo pilocarpine-induced seizure [16] (Table 1).

### 3.4. Hypoglycemia 

#### 3.4.1. NOX Induced Neuronal Death after Hypoglycemia 

The cerebral cortex and hippocampus are the most vulnerable areas to hypoglycemic damage. The damage is driven not only by energy metabolic aspects but also by multiple synaptic changes, mainly arising from glutamate excitotoxicity. Hypoglycemic neuronal death also involves zinc-accumulation-associated damage. Hyperglycemia induces excess glutamate, aspartate, and zinc accumulation. Moreover, postsynaptic zinc accumulation results in reactive oxygen species (ROS) production by mitochondria or NADPH oxidase, leading to DNA damage and the activation of poly (ADP-ribose) polymerase-1 (PARP-1), mitochondrial permeability transition (MPT), and cell death [8,68]. After severe hypoglycemia, microglial activation contributes to neuronal cell damage by releasing several neurotoxic substances, including superoxide and nitric oxide [39,69]. The relationship between glycogen and NOX lies with hexose monophosphate and glucose reperfusion (GR). Temporal hyperglycemia during reperfusion can cause both functional hypoglycemia and the activation of NADPH oxidase [57]. The latter is the main source of ROS during GR, as Suh et al. have shown [39] (Table 1).

#### 3.4.2. Neuroprotective Effect of NOX Inhibition on Hypoglycemia

Considering the pathophysiological connection between NOX and hypoglycemia, it is perhaps unsurprising that the inhibition of NOX alleviates the impact of hypoglycemia. The p47phox-deficient mice that lack NOX protein assembly showed less ROS production after hypoglycemia with ethidium fluorescence staining. The same study provided evidence that zinc chelation also ameliorates neuronal death. As the influx of excessive zinc from hypoglycemia activates NOX, NOX inhibition may have therapeutic usefulness in reducing cell death from ROS after hypoglycemia [39].

## 4. NOXs and Neurogenesis

### 4.1. Neurogenesis: A General Understanding

The neurogenesis process is prominent in two parts of the adult brain: the olfactory region of the subventricular zone (SVZ) and the subgranular zone (SGZ) of the hippocampus [71]. Neural stem cells (NSCs) in the dentate gyrus (DG) are related to long-term spatial memory in a pattern separation manner, and NSCs in the SVZ form olfactory memory-associative learning [72,73]. The adult neurogenesis process acts more as a refinement of the existing nervous system than in inducing the bulk storage of information [71]. In this review, neurogenesis in SGZ is mainly described. 

All NSCs exist in the heterogeneous state, which undergoes both symmetric division (functional self-renewal) or asymmetric division (one mature to neuron). In both niches, NSCs migrate to certain brain regions and differentiate to immature neurons, showing hyperexcitability and increased susceptibility to synaptic inputs [74]. The process of neurogenesis in SGZ begins with glial fibrillary acidic protein (GFAP)-positive radial glial-like NSCs (type I cells) in SGZ turning into GFAP-negative progenitors (type II cells) in the granular cell layer [75,76,77]. They then migrate as neuroblasts (type III cells), beginning to grow their axon towards CA3 and integrating to preexisting synapses with GABAergic and glutamatergic synaptic inputs. Their maturation as neurons ends as their axon is well connected to synaptic relations in CA3. 

The stage of neurogenesis can be experimentally assessed with prominent biomarkers. Cells that are undergoing DNA replication can be detected with BrdU or H3-thymidine; BrdU-positive cells indicate neuron progenitors. Specifically, the M-phase of mitosis can be tracked by the green fluorescent protein (GFP) or Lac Z, and G1 to S or G2 to M by PCNA. Ki-67 is the marker for dividing cells. SOX gene families are thought of as strong markers for NSC proliferation. GFAP is shown in cells that have astrocytic traits, being an initial marker for neurogenesis as described above. Polysialic acid-neural cell adhesion molecules (PSA-NCAMs) are a strong marker for migration, being a detector in the late stage of the neurogenesis. A similar role can be observed for doublecortin (DCX), neuronal nuclei (NeuN), and proopiomelanocortin (POMC), as they signify immature neurons. NeuroD indicates differentiated cells during neurogenesis, which can be a form of identification of mitotic early neuronal lineage [77,78,79,80]. Taken together, the markers for each step of neurogenesis in SGZ are as follows: type I cells, GFAP+Hes5+ or Nestin+Sox1+Sox2+BLBP+ (brain lipid-binding protein); type II cells, Nestine+Sox1+Sox2+BLBP+ or Mash1+ or Prox1+NeuroD+; type III cells, NeuroD1+DCX+; and immature neurons, DCX or NeuN+ [81].

### 4.2. Balance of NOX Activity and Neurogenesis

Neural stem cells (NSCs) maintain proliferation and differentiation capacity in the brain. NSCs can also initiate self-repair mechanisms in response to neuronal damage. Several studies have confirmed that neural progenitor cells (NPCs) generated in the subventricular region (SVZ) and subgranular zone (SGZ) of the dentate gyrus of the brain migrate to the damaged brain region for neuronal repair. Migrated neuronal progenitor cells contribute to the recovery of cognitive abilities when brain damage occurs [82,83,84,85,86,87,88]. It has been reported that relatively high levels of ROS in SVZ and SGZ are essential for the proliferation and self-renewal ability of NSC/NPCs under uncompromised physiological conditions [89,90]. The effect of ROS derived from NOX on the proliferation and apoptosis of NSC/NPCs after brain injury causes therapeutic confusion as it can negatively affect the repair of the damaged brain in neurodegenerative diseases, including brain injury.

**Figure 2:**
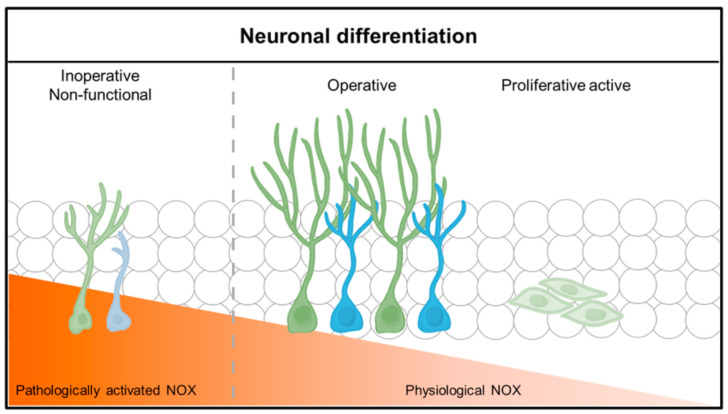
Neuronal differentiation and function according to physiological NADPH oxidase (NOX) activity. Neuronal stem cells (NSCs) differentiate and function into neurons in a physiological or low NADPH oxidase environment. NOX in a physiological state causes lineage differentiation of neuronal stem cells (NSCs), but, under pathological conditions, neuronal stem cells (NSCs) do not differentiate well into neurons or function poorly.

In neuronal disorder studies, such as TBI, HG, GI, and seizure models, it was confirmed that NPCs migrate to the damaged area and differentiate into mature neurons. When single or prolonged neuronal damage occurred repeatedly, SVZ and DG were confirmed to demonstrate transiently increased neurogenesis [84,91,92,93]. However, when NPCs generated in SVZ and SGZ migrate to the damaged area and differentiate into mature neurons, neurons generated in the early stage of damage are often seen to be generated in a structurally and functionally abnormal state, and their survival rate is low [94,95,96,97,98]. Previous studies have suggested that ROS plays an important role in regulating neural stem regeneration and maturation, but uncontrolled high concentrations of ROS in TBI, HG, GI, and seizure and neurodegenerative diseases lead to apoptosis or apoptosis [7,99,100]. During the 4-week follow-up period, it was confirmed that some of the NPCs migrated to the injured site during the first week after injury, which then differentiated into mature neurons. This was observed using the 5-bromo-2’-deoxyuridine (BrdU)/ neuronal nuclei (NeuN) staining of neurons, following long-term BrdU treatment. In NOX-deficient rodents, when NADPH oxidase (NOX) was inhibited during TBI induction, the recovery of motor function was improved, and BrdU/ NeuN staining confirmed active cellular proliferation. One advantage of targeting NOX is that most neonatal neurons in the granular cell layer of DG undergo apoptosis; thus, this approach can be neuroprotective through inhibition of superoxide production, oxidative damage, and weakening neuroinflammation. This suggested that the enhanced neurogenesis in NOX2-KO and NOX inhibiting mice after TBI may contribute to the integration of neonatal neurons in DG by inhibiting the NOX enzyme. By confirming that the number of neurons is also increased, it can be seen that the deletion of NOX contributes to the survival of neurons produced after TBI [96]. Moreover, when apocynin, well known as an inhibitor of NADPH oxidase, was tested in a pilocarpine-induced epilepsy model, the intensity of 4HNE showing reactive oxygen oxidative injury decreased. In addition, it was found that the number of DCX neuronal cells, which identify immature neurons, was increased, and the number of BrdU neurons was also increased. Thus, this intervention is reported to increase the survival rate and differentiation of neuronal progenitor cells into neurons by reducing excessively generated ROS due to neuronal disorder through the deletion or inhibition of NOX. Figure 2 demonstrates that the maintenance of physiological NOX activity after acute brain injury via NOX inhibition or NOX knock out induces physiological neurogenesis.

### 4.3. NOX-Related Neurogenesis after Stroke

Post-stroke analysis has demonstrated that NOX inhibition can be effective in promoting neuronal survival. A study with NOX1 has quantified the magnitude of neurogenesis in AAV-induced NOX1-inhibited mice. There finding suggests that the survival rate of progenitor neurons after MCAO is high in NOX1-inhibited mice in the subventricular zone (SVZ) [56]. Considering the neurogenesis process needs a certain amount of oxidative stress to stay active [101]—but not too much before it is suppressed—, reduction of ROS during the injury setting to keep it within the physiological range should benefit neurogenesis.

### 4.4. NOX-Related Neurogenesis after an Epileptic Seizure

Although epilepsy has shown neurodegenerative traits, it has been found that an epileptic seizure boosts hippocampal neurogenesis. However, this neurogenesis is not considered physiological neurogenesis in which neurons are formed and mature functionally but rather an ectopic response; newborn neurons in seizures are abnormal [93]. Although it was not proved how effective neurogenesis is at establishing functional circuits, our finding indicated that neurogenesis is promoted as cellular death decreases in the hippocampal region when NOX is inhibited. Apocynin-treated mice showed more NeuN+ cells in cornu ammonis 1 and 3, whereas the amount of 4HNE antibody staining was increased compared to the saline-injected sham group. The amount of DCX and BrdU staining was similar after a week from the treatment, but the survival rate was increased at 4 weeks [102].

The programmed cell death (PCD) of neurons occurs in diverse stages during neuronal maturation periods. The apoptosis of migrating neuroblasts occurs with a lack of NMDA receptor expression and activities that increase neural correlation [103]. With this “neurotrophic theory”, in which newborn yet inappropriately developed neurons are eliminated by PCD, it is assumed that NOX inhibition increasing neurogenesis production can indicate that NOX inhibition increases the potential “adequate” neurons that can alleviate memory loss or other complications via supporting post-seizure neurogenesis. 

## 5. Conclusions

There are significant pieces of evidence that indicate that NOX activity is elevated after acute brain injury, and studies with NOX knockout animals and NOX inhibitors support the therapeutic possibility of targeting NOX subunits. Balancing NOX activity has also been shown to improve neurogenesis after acute brain injury. However, there remain many challenges ahead for the discovery of effective and NOX-specific targeting that would yield benefits in acute brain injury while minimizing negative side effects and generating a healthy neuronal micro-environment for ongoing maintenance neurogenesis. 

It is also noteworthy that NOX inhibitors, specifically NOX1 and NOX 4 selective inhibitors, have shown its safety and efficacy in reducing inflammatory markers in clinical trials. As such, pharmacological inhibition of a specific NOX for each neurological disease context may provide a safe and effective therapeutic potential in clinical setting as oxidative stress and inflammatory cascades are denominating, a feature of most acute brain injuries. While addressing these challenges remains a work in progress, the continued advancement of technology and research effort offers hope that the realization of this goal may be achievable in the near future in neurological diseases with unmet medical needs.

## Figures and Tables

**Table 1:** NOX deletion and NOX inhibition effects in animal models. An overview of published studies utilizing rodents.

Disorder	Animal Model	Genetic Manipulation	Treatment	Result	Reference
Stroke				↑ ROS,NF-kB and VEGF	[20,48]
		p47 ^-/-^	Apocynin	↓ Neuron death and superoxide production	[57]
	AAV, MCAO	NOX1 ^-/-^		↓ Peri-infarct, neuron death and activation of astrocytes	[56]
		NOX4 ^-/-^		↓ Ischaemic brain injury	[70]
TBI			Apocynin	↓ p47 phox translocation and neuron death	[61]
Epilepsy	pilocarpine		Apocynin	↓ p47 phox translocation, neuron death and ROS production	[16]
Hypoglycemia	Insulin			↑ Zinc accumulation, ROS production and PARP1 activation	[8,68]
		p47 ^-/-^		↓ ROS production and neuron death	[39]
